# Cancer genetic counseling via telegenetics and telephone: A qualitative study exploring the experience of patients and genetic counselors in an Australian cancer genetics context

**DOI:** 10.1002/jgc4.1982

**Published:** 2024-10-06

**Authors:** Jessica Finney, Verna Fargas, Tina Gonzalez, Natalie Taylor, Claire E. Wakefield, Kathy Tucker, Erin Turbitt, Rachel Williams

**Affiliations:** ^1^ Prince of Wales Hereditary Cancer Centre Prince of Wales Hospital Randwick New South Wales Australia; ^2^ University of Technology Sydney UTS Graduate School of Health Ultimo New South Wales Australia; ^3^ Department of Clinical Genetics Royal North Shore Hospital St Leonards New South Wales Australia; ^4^ School of Population Health, UNSW Medicine and Health UNSW Sydney Kensington New South Wales Australia; ^5^ School of Clinical Medicine, UNSW Medicine & Health UNSW Sydney Kensington New South Wales Australia; ^6^ Kids Cancer Centre Sydney Children's Hospital Randwick New South Wales Australia

**Keywords:** communication, COVID‐19, genetic counseling cancer genetics, telegenetics, telemedicine, telephone

## Abstract

The demand for direct‐to‐patient (DTP) telegenetics (genetics services delivered via videoconferencing) in genetic counseling practice has rapidly increased, particularly since the COVID‐19 pandemic. Recent telegenetics literature is mostly quantitative and not in the Australian context. A qualitative interview study was conducted to address this gap. This research investigated the experiences of patients and genetic counselors (GCs), enrolled in a randomized controlled trial, using telegenetics and telephone for cancer genetic counseling appointments. Twenty‐eight semi‐structured interviews with patients (*n* = 22) and GCs (*n* = 6) were conducted following patient randomization to either a telephone or telegenetics genetic counseling appointment. The interviews explored participant's experiences of telegenetics and compared DTP telegenetics with telephone and in‐person delivery. Codebook thematic analysis was used to develop topic summaries from the data. Patient and GC participants noted positive experiences of telegenetics; with key benefits reported as reduced travel time, time and cost saving, ease, convenience, efficiency, and comfortability. Technical issues and privacy concerns were highlighted as potential disadvantages of telegenetics. All but one patient felt sufficiently emotionally supported while using telegenetics. Telegenetics has both benefits and limitations; however, generally, this cohort found telegenetics to be a suitable and acceptable mode of delivery for genetic counseling with many advantages over in‐person or telephone appointments. Further studies should be conducted to provide evidence for the long‐term implementation of telegenetics, regardless of any future COVID‐19 pandemic lockdown restrictions.

**What is known about this topic?**

Outreach telegenetics has shown many benefits in the genetic counseling field.

**What did this paper add to this topic?**

Direct‐to‐patient telegenetics is an appropriate mode of delivery for genetic counseling for metropolitan patients not just for rural, remote, and regional patients, as has previously been well documented in the literature. This study supports the acceptability of telegenetics in an Australian context and this qualitative approach enriches the claims already found in the literature.

## 1 INTRODUCTION

Genetic counseling is a communication process about genetic testing and risk, giving patients psychosocial support while adapting to genetic information (Resta et al., 2006). Genetic counselors (GCs) working in family cancer clinics (FCCs) assess their patients' cancer risk, guide management, and arrange genetic testing if indicated while providing psychosocial support (Australian Institute of Health and Welfare, 2019; Hampel et al., 2015). Improving access to genetic counseling can help reduce the physical and psychological burden of cancer on families and society; however, there are known barriers to attendance for genetic counseling appointments (Vogel et al., 2018). Telegenetics is a possible solution to reduce barriers and increase acceptability and accessibility of cancer genetic counseling (Danylchuk et al., 2021; Zierhut et al., 2018). The availability and use of telegenetics in cancer genetics, among other healthcare settings, has increased in recent years due to advancement of communication technologies and rapid adoption due to the COVID‐19 pandemic (Gorrie et al., 2021; Murphy et al., 2022). A reported limitation of telegenetics is the inability to perform clinical physical examinations virtually (Brown et al., 2021; Ma et al., 2021). However genetic counseling via telegenetics can be adequately done in the cancer genetics context as a portion of consultations typically do not include any tasks that require in‐person contact with the patient.

In this paper, telehealth is referred to as videoconferencing technology used in the wider health discipline, and telegenetics as videoconferencing is used specifically in the genetics field. Two distinct telegenetics service models exist; (1) outreach telegenetics, which refers to videoconferencing to connect patients accompanied by a GC in a clinical setting with another health professional in a central service (Cohen et al., 2012) and (2) direct‐to‐patient (DTP) telegenetics, which enables a patient who is geographically separated from the GC to use technologies to receive genetic counseling services from home (Diaz & Player, 2020). Prior to the pandemic, the outreach telegenetics model predominated practice and most research to date has focused on this model.

Accumulating evidence indicates high satisfaction and acceptance of telegenetics with reported benefits including reduced travel time and associated costs, convenience, and satisfactory rapport with the clinician (Breen et al., 2022; Buchanan et al., 2015; Dratch et al., 2021; Gorrie et al., 2021; Hilgart et al., 2012; Zilliacus et al., 2010, 2011). This service initiated a pilot study (Gonzalez et al., 2022) before the pandemic to help address the gap in data on DTP telegenetics with metropolitan patients. This work reported no significant differences in patient distress or patients' perceived empathy from the GC between telegenetics, telephone, or in‐person appointments. To date, most current studies focusing on DTP telehealth employ quantitative methods and are largely based overseas. Most studies focus on the United States context (Breen et al., 2022; Dratch et al., 2021; Ma et al., 2021); however, several studies focus on the European context (Coelho et al., 2005; Otten et al., 2016; Pagliazzi et al., 2020; Pestoff et al., 2022; Turchetti et al., 2021), and there are studies on the Asia‐Pacific region (Sim et al., 2021; Tumulak et al., 2021). Given different healthcare systems and GC training pathways (Skirton et al., 2015), present data may not be applicable to Australian practice. Thus, greater depth and nuanced data attainable through qualitative interviews in the Australian context can expand understanding.

Telegenetics continues to be utilized following rapid implementation during the COVID‐19 pandemic (Krueger et al., 2021). The pandemic created an increased demand for videoconferencing technologies conducted from home for social, work, and health contexts. There was an opportunity in Australia to explore the perspectives of patients not captured by previous research, such as metropolitan populations who would not usually opt for the telehealth modality. Data collected in April 2021 by the Australian Bureau of Statistics found that 30% more Australians prefer using online health services than before the pandemic (Australian Bureau of Statistics, 2021). The pandemic has also created an opportunity to explore GC's experiences using telehealth exclusively for most referrals.

This sub‐study explores the experiences of patients and GCs using telehealth in cancer genetic counseling field. The aim is to support the evidence that telegenetics is an acceptable mode of delivery and add to the research in the Australian context.

## 2 METHODS

### 2.1 Study design

The sub‐study operated under a larger study, the CONTACT (Consultation via Telehealth to Access Cancer Genetic Counseling) study. The extended CONTACT study employed a mixed‐methods design to evaluate the satisfaction of DTP telehealth in genetic counseling in the FCCs in metropolitan areas. The CONTACT study was planned to compare demographics across three metropolitan sites (one being a metropolitan outreach clinic) and one outreach site. The study aimed to test non‐inferiority of telegenetics relative to standard care in terms of acceptability, satisfaction, and levels of psychological distress to assess the patient participant's preferred treatment modalities in the context of cancer genetic counseling. A pilot study for the CONTACT study has been completed and showed acceptability of telehealth, high perceived GC empathy, and high satisfaction scores (Gonzalez et al., 2022).

This sub‐study described here aimed to explore the experiences of patients and GCs in these FCCs, using telehealth during COVID‐19. The present study was a complementary qualitative interview study, which added depth and richness to the data that may not be captured by quantitative measures. Qualitative data in conjunction with quantitative data can help bridge the gap between research and application to inform the next steps of implementation of telehealth in real‐world settings (Morris et al., 2011).

### 2.2 Sample and recruitment

The study sample included adults who were referred and attended a risk assessment cancer genetic counseling appointment at one of two metropolitan FCCs in Sydney, New South Wales as part of the CONTACT study, from February 2021 to April 2022. Twenty‐nine patients were invited to participate in the study; seven declined. All six GCs who provided care for these patients through the CONTACT study were invited to participate in this study, but none declined.

Patient and GC participant samples were recruited through purposive (Bernard, 2002) and convenience (Dörnyei, 2007) sampling to enable recruitment of a broad sample population. Patients were invited to participate at the point of triage through a telephone call conducted by a GC or an administrative assistant at the FCC, to which the patient was referred. Patients were invited to join the overall CONTACT study and subsequently to opt‐in for an additional 30‐min interview to occur 1–2 weeks following their initial appointment. At the time of consenting to the overall CONTACT study, patients were randomized to either a telephone or telegenetics appointment using the videoconferencing platform, PEXIP. GCs working at one of the two FCCs were invited to participate in the CONTACT study via email invitation. Henceforth patient participants are referred to as patients and GC participants are referred to as GCs.

Patients for this study were adults referred to a cancer genetics clinic who required a risk assessment to assess cancer risk and eligibility for genetic testing, i.e., they did not qualify for genetic testing based on the referral alone, and further information such as family history or pathology was needed.

The inclusion criteria were as follows: over 18 years old and fluent in English, have the capacity to provide informed consent, and have access to technology (specifically a telephone for the interview). The recruitment was not limited to a specific type of cancer. Any patients who had complex psychological difficulties or suicidal/psychotic symptoms were excluded from the study. These were identified either in the referral as an individual with a known psychiatric disorder or in direct exploration by the senior GC prior to the appointment in response to the patient's initial Kessler Psychological Distress Score, K10 score, greater than or equal to 25, indicating moderate psychological distress (Kessler et al., 2002). Those requiring urgent genetic counseling appointments were also excluded, with appointments deemed urgent if a genetic test result would influence a patient's upcoming treatment and or surgical plan, or for imminent fertility planning.

The inclusion criteria for GCs participating in the CONTACT study were English speaking, able to consent and participate, agreed to be contacted about the study, and available for telephone interviews within the time frame of the study.

### 2.3 Data collection

The interview schedule (see Appendix S3) was developed from interview questions reported in the literature (Clay‐Williams et al., 2017), along with expert input from members of the research team who had experience with previous telegenetics studies. Topics covered in the interviews included feelings before the counseling appointment, what the patient needed to prepare for the appointment, how they felt during the appointment, and their connection with the GC/patient. The participants were asked to reflect on benefits and difficulties of their mode of delivery.

JF and VF (student researchers) completed the interviews over two different time points using the same interview guide. JF completed 11 patient interviews in February/March 2021 and VF completed 10 patient interviews and 6 GC interviews in March/April 2022. The researchers conducted semi‐structured phone interviews with patients approximately 1–2 weeks after the patient's initial appointment to explore their views and experience with their specific mode of delivery. The researchers had no prior contact with the patients prior to the phone call and no external persons were present during the interviews. The researchers were either located in a private room in their homes or the hospital to conduct the interviews.

The interviews were audio recorded and then transcribed using external transcription services. There were no repeat interviews carried out. The researchers manually reviewed the transcriptions against the recorded interviews for accuracy. Neither patients nor GC participants had further involvement after their interview.

### 2.4 Data analysis

Codebook thematic analysis was used (Boyatzis, 1998). There are three different types of thematic analysis referred to as coding reliability, codebook, and reflexive (Braun & Clarke, 2022). Codebook thematic analysis involves the use of a structured codebook, which was developed from interview data and with input from the research team. The codebook then guided analysis of the interviews, though codes were iteratively added as they were developed from the data. Codebook thematic analysis was used, as it afforded us a more structured approach to analysis, which was important with multiple coders with a range of experience, from novice to experienced, in qualitative analysis. Themes were conceptualized as topic summaries. We used a codebook to code the data and conceptualized themes as topic summaries. The use of a codebook and topic summaries aligns more with a positivist approach to analysis, though the researchers were reflexive in their coding and considered researcher subjectivity as an asset.

Specifically, codebook thematic analysis involved first coding the data using NVivo 12 Pro software, converting the raw data into labels (codes) that categorized, summarized, and accounted for each piece of data. Topic summaries were then developed and described by drawing together codes and categories to answer the research question. The topic summaries were next revised, refined, and named (Castleberry & Nolen, 2018).

The researchers coded the interview data from the interviews they each conducted, receiving support from RW (senior GC) and TG (GC) who co‐coded five interviews, and ET (experienced qualitative researcher) who provided overall method guidance. The coded data from all interviews was then combined. Coding and analysis were discussed during regular meetings with the research team, and any coding discrepancies were resolved in these meetings. The transcripts were coded until topic saturation was reached; this was apparent when no new topic summaries were developed and sufficient information was obtained to answer the research question (Green & Thorogood, 2004).

## 3 RESULTS

Twenty‐two adult patients who saw one of six GCs in one of two hospitals in New South Wales for an initial genetic counseling appointment were interviewed. Thirteen of the patients were assigned a telegenetics appointment and nine were assigned a telephone appointment. Patient's ages ranged from 24 to 82 years (see Tables 1 and 2 for demographics). The patients all lived in the Sydney metropolitan area.

**TABLE 1:** Overall demographic characteristics of patients.

Demographic	*n* ^a^	(%)
Age		
0–30	2	9.1
30–39	7	31.8
40–49	7	31.8
50–59	3	13.6
60–69	2	9.1
70–79	0	0.0
80+	1	4.6
Sex		
Female	18	81.8
Male	4	18.2
Mode of appointment		
Telegenetic	13	59.1
Telephone	9	40.9
Hospital		
1	19	86.4
2	3	13.6
Tumor status		
Affected^b^	9	40.9
Unaffected	13	59.1

^a^ *N* = 22.

^b^ 4 breast cancer, 3 polyps, 1 colorectal, 1 multiple primary tumors (four).

**TABLE 2:** Specific characteristics of patients.

Patient number	Appointment type	Sex	Age (documented at time of appointment)	Hospital (where patient was referred to FCC)
1	Telegenetics	Female	67	2
2	Telegenetics	Female	47	2
3	Telegenetics	Male	49	1
4	Telephone	Male	66	1
5	Telephone	Male	82	1
6	Telephone	Female	52	1
7	Telephone	Female	48	1
8	Telegenetics	Female	57	1
9	Telegenetics	Female	31	1
10	Telegenetics	Female	46	1
11	Telegenetics	Female	45	1
12	Telegenetics	Female	34	1
13	Telegenetics	Male	36	1
14	Telephone	Female	37	1
15	Telegenetics	Female	24	1
16	Telegenetics	Female	40	1
17	Telephone	Female	38	1
18	Telephone	Female	31	1
19	Telegenetics	Female	54	1
20	Telephone	Female	25	1
21	Telephone	Female	36	1
22	Telegenetics	Female	49	2

All GCs (*n* = 6) participating in the CONTACT study were interviewed. This included five GCs from one FCC and one from the second participating FCC. GCs had a range of years of experience from <1 year to over 20 years with an approximate mean of 8 years. All were female. GCs self‐reported a range of comfort with information technology (IT) from “not very good” to “fairly high” with most GCs ranking themselves as average or just below.

Six topic summaries that followed the interview schedule were developed. These include overall satisfaction and suitability of telegenetics and telephone, benefits of telegenetics and telephone, privacy concerns, telegenetics versus telephone; visual cues and engagement as well as rapport, social presence, and emotional support.

### 3.1 Overall satisfaction and suitability of telegenetics and telephone

Patients spoke positively about their mode of delivery and their overall genetic counseling experience. The patients agreed that the GC was “super easy” (P20) to talk to, “really engaging” (P21), and that their main concerns were addressed. The majority of the patients stated that they would choose telegenetics or telephone appointments instead of in‐person if they had been offered a choice for that initial appointment.

It was really good. [GC name] went into lots of detail and explained everything even beyond what she needed to. And she took her time. She wasn't in a rush and I could ask her any questions. Yeah it was really, really good. (P11)

All GCs agreed that telegenetics was a suitable mode of delivery for hereditary genetic counseling services:

You're giving people a lot of information with cancer and collecting a lot of information, and for that, I think [telegenetics] is perfectly suitable. (GC06)

### 3.2 Benefits of telegenetics and telephone

Patients highlighted many benefits of telegenetics and telephone appointments compared to in‐person appointments. These included reduced travel time, travel expenses, time‐saving, ease, convenience, and efficiency of telegenetics/telephone. Many patients described being able to attend their appointment more easily, which could increase equity of access, reducing barriers of attendance for individuals with busy lifestyles, those in caring roles, and those for whom the distance to come to the hospital is a barrier, who live further from the hospital.

I didn't have to take any time off, it was just easy for me to just jump on [in the] middle of my workday and just get it done […] rather than having to get out, get to the car, go and see someone. (P21)

That is one big advantage I guess is the parking […] finding parking close enough and not paying a fortune. (P2)

Patients emphasized the comfort of being in their own homes or a familiar environment. Telegenetics/telephone appointments were described as less stressful than in‐person appointments; “less stressful and more efficient” (P7).

The GCs agreed that the main benefit of telegenetics was the convenience for patients:

The main feedback that I've been getting is the convenience factor […] for many people and I have to say this is the same for myself […] we're very busy. They don't have to take a sick day from work to go to a medical appointment. (GC05)

GCs shared that the noted benefits of telegenetics increase the FCC's “equity of access or accessibility” (GC06), enabling an increase in the “level of service that we can provide and the number of people that we can provide it to.” (GC06). Telegenetics enables continuity of care allowing patients access to their services from anywhere:

I just had a patient now, he said they're moving overseas for 2 years. If they needed to talk to us, we could still do it. (GC04)

### 3.3 Technological aspects of telegenetics

Patients shared the view that telegenetics using the PEXIP platform was simple and easy to use from a technological perspective. Some described their confidence in information technology (IT) as low but did not have any issues with telegenetics, as they had family members to help, found it easy to click on the link or follow the instructions, or because they were using familiar devices. Most of the participants have had some experience with videoconferencing software due to the COVID‐19 pandemic, which increased their comfort with using telegenetics.

Usually I hate technology, but I was comfortable at home with my laptop. (P2)

I am pretty old school, but I have had to learn how to follow instructions, so it was all good. (P3)

There were minimal technical issues mentioned. Patients discussed that when such issues did occur, they were usually able to overcome them or were not bothered by them. One patient had a technical fault and when asked if this concerned him, he responded:

No, not at all. You know when you have a cancer scare, nothing really worries you. With what I have gone through nothing is that big, just microscopic issues that bounce off me, nothing to be stressed over. (P3)

Patients noted that they felt assured by the GC's ability to provide technological support if needed.

I had so much confidence in [GC name]. I knew she would have replied and helped me for 3 h if something had gone wrong. She would have helped, and we would have carried on, I have absolutely no doubt. (P4)

One patient, who had a telephone appointment, mentioned she believed the GC would have been able to assist her if she had been assigned a telegenetics appointment; however, she would have felt uncomfortable if she needed help. When asked what support she would have liked to have a telegenetics appointment, she responded:

Just make sure the person on the other end had lots of patience and time. I am sure they would have been able to tell me which buttons to push and if I had my camera upside down. However, if I was meeting them for the first time though I would have felt like a right dummy. (P6)

Patients suggested that sending a link to the videoconferencing call on the day of the consultation would improve their experience. Otherwise, most patients could not think of any more technological support they would want from the FCC. One of the patients suggested that the lighting and the GC's position in the frame could be improved.

There was a lot of head space and bad lighting and therefore it is actually harder to communicate with the counsellor because you can't really see who they are as a person. (P10)

GCs reported that telegenetics was easy to use. Half the GCs agreed telegenetics was “very, very easy” (GC02) when asked to compare it to in‐person. The GCs associated varying degrees of stress with telegenetics noting the potential for technical error.

[Telegenetics], it's a bit of stress in the sense that I don't know if it's going to work…it's the stress of losing time. (GC02)

Most of the GCs agreed that the telephone was less stressful than telegenetics due to the lack of technical issues, “a phone you just turn it on. You talk to them, and it works.” (GC03).

However, GCs noted that they have learnt to adapt and become more confident in their ability to self‐troubleshoot any technical issues.

I think over time, though, in the last 2 years, it's become general practice. I guess we're getting better at it, and it's become our new norm. (GC04)

### 3.4 Privacy concerns with telegenetics

Both patients and GCs discussed privacy concerns. One patient was not comfortable with telegenetics due to a prior adverse experience of cyberstalking.

My existing overarching thing is extreme prejudice against online. Not because I don't like the actual appointment but because I don't like the idea that somebody is listening in to my appointment. (P10)

However, for others, privacy was not a major concern and would not stop them from using telegenetics:

I guess I'm aware that there's potentially privacy things, but it's not a significant worry for me. (P13)

One GC expressed that it was difficult to ensure patient privacy when the patient can join a consultation while in a public place:

We have gone to lengths to try and guarantee their privacy, and here they are in a cafe, and we're visible. And they're obviously going to be heard at the tables around them. (GC01)

Another privacy concern highlighted by the GCs was not always knowing who else was in the room with patients:

Sometimes I do have that slight worry that if there is someone else outside of the room that I can't see. I'm wondering what influence they have over what's being discussed from the patient's point of view. (GC06)

One GC talked about their experience with a technical glitch with the platform that had the potential to breach patient privacy because patients were entering incorrect virtual rooms that may have had another appointment occurring, rather than the virtual room they had been assigned.

### 3.5 Telegenetics compared to telephone: Visual cues and engagement

Patients shared that they felt telegenetics was advantageous over telephone due to visuals of the other person, picking up on body language and visual cues as well as viewing documents and other visual aids.

She could actually see the things that maybe I felt more difficult to process. (P22)

Patients mentioned that telegenetics is preferable to telephone as telegenetics offers less distraction, and they “pay attention more if I'm on video.” (P16).

One patient highlighted a benefit of telephone over telegenetics was the ability to be comfortable to move around, which helped them be engaged, “I do prefer the phone because I can put my earphones in and I can move around if I want to, it's just a bit easier” (P20).

A lack of visuals for the telephone was noted by some patients as a positive.

I am happy just to have a phone call, I feel a bit weird when someone's looking at me. (P7)

One patient explained that a lack of visuals is better than poor visuals, as it allows them to envision the appointment in a more positive way.

If the camera connection was poor, then in fact I would prefer a phone call because then my imagination can fill in the gaps rather than feeling somewhat alienated by the doctor because it is a dodgy way they have set up their visuals. (P10)

The majority of GCs highlighted that the visual component of telegenetics was a benefit, contributing to better rapport and engagement:

It's just helped me in terms of building that rapport and knowing that the patient is still with me during that discussion, and it helped me to know for sure that we're actually moving together at the right pace. (GC05)

GCs discussed that the visual component of telegenetics also enabled them to share resources better compared to the telephone, where you would just have to describe it to the patient:

I can show them the consent form and get a better response from them. Whereas over the phone, I mean, you just go through it and I'm not really sure, they might say ‘yes, I understood’, but I'm not really sure. (GC02)

Another GC noted a challenge of telephone consultations was the lack of visual components contributing to a lack of patient engagement.

In my general experiences, they completely don't value that that's a legitimate appointment with the clinician. (GC01)

GCs reported that they had to adapt their practice over telegenetics and the telephone. They had to “do more check‐ins” (GC01) than in‐person and explain clearly what they are doing. The GCs noted they would use their hands more for explanations over telegenetics.

I will often show them at the very start after drawing some people and just say ‘I'm just drawing this family tree’ […] and just explain why I've got my head down. (GC03)

### 3.6 Rapport, social presence, and emotional support

Patients noted positive relationships and rapport with the GC and felt the GC was able to convey bedside manner and empathy over the screen or telephone. Patients highlighted that other healthcare professionals may not be able to build this connection; however, due to the counselor's personality traits, patients reflected that this was not an issue.

The GC was very pleasant and empathetic, and she could show you that through the screen. But if the person doesn't have it, then this can be quite missing over the internet. If they don't have those qualities, it doesn't matter if you are there in‐person or not. (P10)

Patients discussed the difference in “presence” between an in‐person appointment compared to a telegenetics/telephone appointment. Patients talked about feeling a strong relationship with the GC but still felt like an in‐person appointment would have been more personable.

I think that it will always be much more personal when you are with the person, it just feels more comfortable and in‐person is always better. (P1)

This social presence wasn't always referred to as being better or worse but rather a “different energy” (P3).

There is this intangible connection that you can have either positively or negatively by sitting in the same room as somebody that is harder or impossible to achieve when you are on a telehealth [telegenetics] conference. (P10)

The majority of the patients felt sufficiently emotionally supported via the telephone or telegenetics. Some patients felt adequately supported in their current state; however, felt that if more emotional support was needed due to a change in their situation or increased stress levels than telegenetics /telephone would not suffice.

[Telegenetics] is not going to be for everyone and it depends on the subject matter and where individuals are at emotionally. It was fine for me, but I could imagine there are people who are very stressed and in‐person is going to be better for them. If I was actually diagnosed with cancer then I suspect I would want an in‐person appointment. (P8)

One of the patients explained that she would have elaborated on the psychosocial aspects of the appointment if it had been in‐person. She didn't feel comfortable enough to share her past experience of being stalked online and the emotional impact of this.

I don't think they are as effective on an emotional level because I probably would have explained what my issues were a little more closely had I been in a room. (P10)

Perceptions of patient engagement ranged across the GCs. Half the GCs commented that there was increased patient engagement over telegenetics, and a couple expanded to comment that the ability of patients to “have a medical appointment in their own time and in their own space” (GC06) enables them to be more forthcoming, increasing rapport, and engagement:

I think the patients feel more comfortable for those recovering from surgery. You know they don't have to feel worn out having to come to the hospital. They can be comfortable in their lounge or in their bed if they need […] it's more relaxed and I think that gives us a more engaged patient. (GC01)

The other half of GCs perceived “less chit chat” (GC04), which then makes it “harder to build rapport with them” (GC04). One expanded further to comment that the lack of patient engagement was frustrating.

It is frustrating for me as a clinician to have that unconditional positive regard for a patient when I feel that they're not even engaged with me or what I'm trying to do for them. (GC05)

The loss of being in the same room as the patient was noted by half the GCs as a challenge associated with telegenetics that would impact the suitability of its use for specific consultations. Some GCs identified that in highly emotional consultation, they felt “a bit powerless” (GC06) and telegenetics lacks “that touch that gives them comfort” (GC02). They suggested that they could offer some comforting touch or tissues in‐person, and they thought it was difficult “thinking that they are then at home by themselves” (GC03).

## 4 DISCUSSION

This cohort expressed overall positive experiences adding to existing data showing that telegenetics and telephone are suitable modes of delivery for genetic counseling. The benefits and high satisfaction reported by patients and GCs in this study are consistent with the literature. Previous studies have found patients had positive experiences with telegenetics and felt equally satisfied with telegenetics as an in‐person appointment (Boothe & Kaplan, 2018; Kubendran et al., 2017; Mette et al., 2016; Otten et al., 2016). Likewise, the suitability of telegenetics expressed by all GCs in this study aligns with previous reports of high acceptance and satisfaction with videoconferencing amongst GCs (Ma et al., 2021; Zierhut et al., 2018). GC's acceptance of telegenetics is driven by increased confidence and ability to adapt as well as increased equity of access, readability of nonverbal cues, and ability to build rapport over the screen.

Convenience and reduced travel were the primary benefits for participants who chose telehealth as their top preference for future genetic counseling appointments. GCs similarly listed the main benefit of telehealth and telephone appointments as the convenience for the patient, as it enables FCCs to have access to more patients. Convenience and reduced travel and costs are well‐researched benefits of telehealth (Bradbury et al., 2016; Kubendran et al., 2017; Zilliacus et al., 2010). These prior studies focused on regional and interstate communities and different service models of telehealth. This study suggests that metropolitan patients close to their genetic services also appreciate the convenience and reduced travel time.

Drawbacks of telegenetics in this study included technical issues, privacy concerns, and lowered patient engagement. While technical issues are a well‐established drawback associated with telegenetics (Cohen et al., 2016; Mills et al., 2021; Terry et al., 2019), the notion of privacy concern is sparse in the literature. A study by Bradbury et al. (2016) found that one‐quarter of their cohort raised confidentiality as a concern with telegenetics. Pestoff et al. (2019) noted clinician concern about patient safety and the inability to provide equivalent support when using telegenetics compared to in‐person appointments; however, this was pre‐covid pandemic when clinicians were less experienced in telegenetics. Although technical issues occurred in this study, most patients and/or GCs were able to resolve them without assistance. This could be due to the increased use of telegenetics and improvements to this technology since the COVID‐19 pandemic leading to more familiarity and confidence.

The main difference when comparing telegenetics and telephone appointments was visual cues and how this relates to engagement. It has been reported that telegenetics, and particularly telephone calls, resulted in less engagement from the patient and the clinician, compared to in‐person (Thomas et al., 2022). However, in this study patients did not note less engagement from the GC, supporting the idea that GCs have adapted to this mode of service delivery. The majority of the study's GCs perceived the ability to see patients and read nonverbal cues enabled increased patient engagement and some noted a lack of engagement over the telephone. The GCs who perceived a decreased patient engagement echoed the perceptions of GCs in earlier studies who had less rapport and felt a limitation in providing a quality service to patients, challenging their satisfaction (Otten et al., 2016; Zierhut et al., 2018).

Most of the patients felt that telegenetics/telephone met their psychosocial needs at the time. This is reflected in past studies showing that psychological outcomes do not differ between patients attending in‐person versus telegenetics appointments (Otten et al., 2016; Solomons et al., 2018). All but one patient in this study felt sufficiently emotionally supported throughout their telegenetics/telephone consultation; despite reporting a positive relationship toward the GC, she did not feel sufficiently supported to explore psychosocial aspects in the appointment. A similar finding was reported by Zilliacus et al. (2010), which concluded that telegenetics may be less suitable for more complex cases. This study offers some support to this, with a few patients positing that had their situation been more severe or complex, telegenetics may not be sufficient. Taken together, these findings indicate the patient's level of psychosocial needs that should be taken into consideration when deciding on a mode of delivery and assessed on a case‐by‐case basis. The patient's preference as well as the appointment type (e.g., intake versus result giving) should also be considered.

### 4.1 Limitations

The researchers were key instruments of data collection and acknowledged their prior experiences, knowledge, and characteristics can influence decisions about interviewing methods and analysis (Clarke & Braun, 2021). The researchers acknowledge that having two separate interviewers as well as two different time points could bring differing interviewing techniques, interpretation of the results, and differing patient views. JF and VF kept a reflexive journal throughout the interview and data analysis stages to manage reflection on their influence.

As randomization to either a telegenetics or telephone appointment was a component of the study design, patients who expressed a strong preference toward one mode of delivery often did not join the CONTACT study. However, due to the COVID‐19 health advice and related hospital policy, the participating hospitals did not typically offer initial in‐person appointments outside of the study during this time.

The patients were also reflecting on their first genetic counseling consultation and therefore did not have other experiences with other modes of delivery in genetic counseling to compare.

As the patient and GC samples were biased toward one FCC, it is possible that the clinical process and use of telegenetics may not mirror experiences at other FCCs. Moreover, both the patient and GC cohort displayed limited cultural diversity, with all patients having English as their primary language. There also was limited demographic data collected for the patients and the inclusion criteria were narrow due to the selection from the overall CONTACT study population. Data was also not gathered to compare this patient population to the overall CONTACT study. Further research toward culturally and linguistically diverse patients' experiences and the use of interpreters through telegenetics genetic counseling is needed. Now the workforce is more experienced in telegenetics, future randomized studies comparing genetic counseling appointments in‐person and telegenetics will inform future practice.

## 5 CONCLUSION

The COVID‐19 pandemic has impacted genetic counseling service delivery, rapidly shifting standard care to telegenetics during this time. As these systems are established, with largely positive feedback from both patients and GCs, telegenetics will likely remain common practice. This study adds to the body of evidence demonstrating that telegenetics is not only appropriate but can be advantageous for genetic counseling appointments in the cancer genetics field in Australia. By reducing barriers, improving accessibility, and increasing acceptance of the technology, this data helps support the integration and long‐term use of telegenetics in genetic counseling. Future research into the needs of diverse communities will strengthen the understating of the broader acceptance and suitability of telegenetics for a wide range of genetic counseling scenarios as other areas of genetics have differing needs such as physical examinations.

## AUTHOR CONTRIBUTIONS

Author, Jessica Finney confirms she had full access to all the data in the study and takes responsibility for the integrity of the data and the accuracy of the data analysis. Authors Rachel Williams, Kathy Tucker, Natalie Taylor, and Claire E. Wakefield were involved in the conception and design of the study. Tina Gonzalez was involved in the qualitative design of the study. Interviews were conducted by Jessica Finney and Verna Fargas and data analysis and interpretation were performed by Jessica Finney, Verna Fargas, Tina Gonzalez, Rachel Williams, and Erin Turbitt. All of the authors gave final approval for this version to be published and agreed to be accountable for all aspects of the work in ensuring that questions related to the accuracy or integrity of any part of the work are appropriately investigated and resolved.

## FUNDING INFORMATION

This study was funded by a Translational Cancer Research Network (TCRN) grant. The TCRN is funded by the Cancer Institute NSW through a Translational Cancer. Research Centre grant (13/TRC/1‐03). C.W. was supported by a Career Development Fellowship and an Investigator Grant from the National Health and Medical Research Council of Australia (1143767 and 2008300 respectively).

## CONFLICT OF INTEREST STATEMENT

The authors, J.F., V.F., T.G., R.W., E.T., N.T., C.E.W, and K.T., all declared that they have no conflict of interest.

## ETHICS STATEMENT

Human studies and informed consent: The study was approved by the South East Sydney Local Health District Human Research Ethics Committee (2019/ETH13294) and ratified by the UTS Human Research Ethics Committee (ETH20‐5598). Informed consent was obtained from all participants and all data was de‐identified.

Animal studies: No non‐human animal studies were carried outby the authors for this article.

## Supporting information


Appendix S1

Appendix S2

Appendix S3

## Data Availability

The data are not publicly available due to privacy or ethical restrictions. Further information is available in the Appendices [Link], [Link], [Link] or by contacting the corresponding author.
