# Supplementary material for: Cancer genetic counseling via telegenetics and telephone: A qualitative study exploring the experience of patients and genetic counselors in an Australian cancer genetics context
Source: J Genet Couns. 2024 Oct 6;34(2):e1982. doi: 10.1002/jgc4.1982 (PMC11953582; doi:10.1002/jgc4.1982)
Supplement: Supplementary file 1 — Appendix S1 [file JGC4-34-0-s001.docx]

| **Code** | **Description** | **Summary** | **Quotes** |
| --- | --- | --- | --- |
| TH Benefit - Continuity of care | Benefit of TH is because of ability to access anywhere and the convenience to fit around work schedules, can continue care | 2 GCs commented on ability to continue to offer care using TH that would not be possible without it | " I just had a patient now he said they're moving overseas for two years. If they needed to talk to us, we could still do it." - GC 4. "One of the benefits of that we could continue doing that through COVID as well, that we could actually do those things from home" - GC3 |
| TH Benefit - Convenience | The benefit of TH is that for patients, it makes GC appointments more convenient | 4 GCs commented how TH is more convenient for patients to access GC services | “I think allowing people with flexibility to not have to take a half day out of their workday to come into the hospital. It's given a lot more flexibility for mums of young children who are juggling, you know. Cranky children or sleep times or you know dropping off the older child and you know, running around kids' appointments or just trying to keep the household running, all those sorts of things. So from that perspective, I think it's worked really well. Probably the biggest thing I've noticed is their appreciation from workers who don't work in [hospitals area] but in fact has found this very useful.”– GC1.  “They certainly appreciate the convenience of being able to you know have a medical appointment in the in their own time and in their own space. I mean a woman yesterday she said you know, if I had to come into the hospital. I would have had to have taken a day off work, whereas here I can find a quiet spot at work and I can do it from work, you know so much more convenient.” – GC6 |
| TH Benefit - Equity of access | TH allows for more patients to access GC services that would otherwise be limited due to distance and time | 2 GCs made direct comment on how TH increase equity of access | “I think it is, it's a very handy way of talking to patients, especially if they do have far to travel so we can speak to people who are further away than we wouldn't normally.” - GC4.  “I just think it increases our whether you want to call it equity of access or accessibility or you know, it just makes it, makes it better. The level of service that we can provide and the number of people that we can provide it to.” – GC6 |
| Demographics - GC IT Literacy | How the GCS described their own IT literacy/confidence | 3 GCs described their IT skills as average, 1 above average, 1 excellent, 1 below average | n/a |
| Demographics - Previous TH experience | GCs previous telehealth/telemedicine experience | 3 had no previous TH experience, 1 had a telemedicine experience, 2 had some exposure to TH before CONTACT, one in general genetics | n/a |
| TH - GC adapting to TH - IT support | Times when GC had to call formal IT support or peer support | 3 GC reported experiences of calling formal IT support, however these experiences are associated with being time consuming and not always answering the issue and one said that their first port of call was peers for It support. | “So I ended up calling the IT service desk and it was, you know, settings within that patient smartphone…Where even though it said that they had allowed access they actually hadn't allowed them access to their microphone and their camera. I thought we've resolved it, we got the camera to work but then we lost sound and so I wasn't going to call tech services back. I just said look, let's just continue on a phone because by then I'd lost 20 minutes of the appointment time.” – GC 5 |
| TH - GC adapting to TH - troubleshooting | GCs commenting on troubleshooting process | 5 GCs reported experiences of troubleshooting to resolve technical issues during appointments. And whilst associate frustration and the time-consuming nature, over the two years of using telehealth they feel confident to troubleshoot before calling on other support or continuing on with the appointment. | "I think that maybe through experience, both over time, we've become used to if something isn't working, it hasn't meant it's the end of the world. Now it's like, well, righteo, how can we keep this appointment moving?” GC1 |
| TH benefit - Visual prompts | Benefit of TH is you have the visual component that is missed in phone consults e.g., seeing the patient cues | 4 GCs made comment on the visual factor of telehealth is a benefit, mainly contributing to building better rapport | "when it's over telehealth, I just noted that I explained myself much better and quite in detail. Probably that detail wasn't required, but I think it fits, the eye contact that sort of prompt me as well to sort of expand things further. - GC2.  "...I think from that perspective it's just helped me in terms of building that rapport and knowing that the patient is still with me during that during that discussion and it's just helped me to know for sure that we're actually moving together at the right pace." - GC5 |
| TH benefit - Visual prompts | Benefit of TH is you have the visual component that is missed in phone consults e.g. sharing resources | 2 GCs commented on the visual element allows for sharing resources | "That’s where I can show them the consent form, and I can get a better response from them. Whereas over the phone, I mean, you just go through it and I'm not really sure, they might say yes, I understood, but I'm not really sure and I don't read everything because you really don't need to and I don't know, it's, I'm very uncomfortable over the phone. I would much prefer face to face or telehealth.” - GC2 |
| TH - GC adapting to TH | Do GCs change from their usual style/habits when using TH | 1 genetic counsellor did not report any changes to their genetic counselling style. 2 reported that they usually use their hands with explanations and have to adapt that. 3 GCs made referenced to how they adapt to the pedigree drawing component in their sessions. (Link to sharing resources). 2 reported less rapport because of barrier of being in the room – one inability to just as easily share resources. | “I think they were sitting next to me or you know I would have a big folder of resources perhaps that I might just say, hey, look at this picture or you know, look at me drawing your pedigree or you know you get a little bit of that sort of, a little bit less of that sort of interaction.” – Gc 6 |
| Impact of COVID | Gcs mentioned how COVID had impacted their use of TH | 5 Genetic counsellors acknowledged that the influence of COVID-19 has meant that telehealth has become the new norm for participants but also for themselves. And whilst there are technology barriers, participant and clinicians are accepting and are not disruptive to the appointment. | “I think over time though, in the last two years it's become general practice. I guess we're getting better at it, and it's become our new norm, so it'll be hard and out when when things change and we eventually get patients back in. It will feel weird to us to see a lot of patients face-to-face 'cause the norm is now telehealth.” – GC4 |
| Experience with interpreters | GCs experiences with using interpreters over phone and TH | There were varied experiences of working with interpreters, 3 GCs preferred the phone over TH because of the increased potential for error and the increase time pressure of the interpreter's availability. 2 had positive experiences were TH increased engagement and 1 GC found no difference when using TH compared to phone | *“I don't feel that they've if I that I've been adequately able to sort of show them those forms via telehealth and then I rely on yet another person, perhaps a family member to show them how to complete the consent form I, you know, I always worry if that's an extra bound, an extra barrier of making sure that that's a, you know, an effective translation at the very end there once they've actually received the forms that I've sent through…the extra barrier with the having an interpreter present is again, they often have a very strict timeline, so if you're wasting 20 minutes trying to troubleshoot with IT support to get that patient connected, you are losing really valuable time.” – GC5* |
| TH - suitability of TH | What contexts is TH suitable | All GCs agree that TH was suitable for cancer GC and sees the utility of it in the future because of the convenience and increase accessibility benefits. 2 GCs reported that for situations that are highly emotional, TH can be limiting and practically may not be suitable if more support is needed. 3 GCs commented that when physical examinations are needed, TH would not be suitable. | *“When someone is really upset, its difficult, “thinking it that they are then at home by themselves and that sort of thing.” - GC 3. “The one limitation is of course, things like clinical examinations or a little bit more and you know which have done sometimes in cancer genetics but not necessarily always at that first appointment...” GC 5* |
| TH Challenge - loss of physical element | The loss of being in the same room as the patient presents challenges to GCs | 3 GCs mentioned how loss of physical element of a face-to-face consult resulted in either a loss of body language or the loss of body touch was inhibiting | "...some benefits of still being in the same room with a person you know it's a much more immediate recognition of any signs that they could be you know their, their physical cues could have changed you, you don't necessarily have to have a really obvious change. You can pick up things that are a little bit more subtle when you're in the same room with them." - Gc5. "if someone is showing a great deal of emotion on the other side of you know on on telehealth like they're crying or something. And perhaps if I was in the room with them I would attempt to you know comfort them in a probably a different way. You know it's very hard to comfort someone you know. You sort of feel like you're a bit powerless to do anything" - GC6 |
| TH Challenge | Other challenges of using TH not captured in other codes | 1 GC commented on the longer process of TH, 2 commented on you are dependent on the patient in particular their IT literacy, 3 said it was harder to share resources | "it's almost like whether not the patient can get training as well as us. We can get everything working in our end, it's just that sometimes that patient, despite having some basic troubleshooting guides that are emailed out through to them, it's often, uhm, well we think that it's often at their end.” – GC 5 |
| TH - Barriers to TH | What prevents GCs from using TH | 3 commented on room availability, 2 mentioned technical difficulties of setting up from home – when they need to work from home in context of COVID restrictions. 1 mentioned the language barrier of the platform could prevent using with members of CALD | "Getting access to a room is probably the biggest barrier for using it.” - GC 1  “The CALD community already have access issues and it would be terrible to think that this technology could work so well for them in terms of not having to rely on one of their children being having to take time off work to drive them into the hospital and navigate them through the hospitals because they can't understand the signs and things like that within the hospital. That's got a lot of potential, but if they're not able to navigate the front screen to get into the virtual waiting room, then that's a problem.” GC 1 |
| Ease of use | GCs comment on how easy TH was to use in comparison to telephone and in-person | Varied responses: 3 GCs ranked TH just as easy as the phone and in-person. 2 GCs gave the ranking of telephone being the easiest, the in-person, and TH as the 3rd easiest. Another GC ranked telephone as easier and in-person and TH the same | “I'd probably put it (Telehealth) third overall because of the potential problems for hiccups” - GC 1 |
| Phone - benefits | When GCs explicitly associated benefits to phone consults | 2 GCs explicitly stated that a benefit of telephone consults was the lack of technical issues. |  |
| Stress of use | GCs on how stressful is using TH | Majority of GCs (5/6) associated some stress with telehealth because of the potential for technical errors. The exception said frustrating was a better word rather than stressful | “I would say it's frustrating rather than stressful. I think frustrating for better word” – GC 4 “Telehealth it's a bit of stress in the sense that I don’t know if it’s going to work. And then you feel a bit annoyed when the other person is not able to, not annoyed I would say. It's just that the stress of Oh my God I’m losing time. I wish it worked.”- GC 2  “I guess it has a little bit of stress if things don’t work… and I think that little bit of stress has become far more diminished in time because there's been so many occasions that I've got to almost roll with it.”- GC 1 |
| Stress of use - telephone | GCs on how comparing how stressful telephone and in-person is to use versus TH | Majority of GCs (5/6) associated telephone as least stressful with TH ranked same or more stressful or below in-person, naming in-person issues as difficulty finding parking and going to the wrong place. The outlier ranked in-person as most stressful over TH. | *“ whereas if it’s a phone you just turn it on. You talk to them and it works” – GC 3* |
| TH challenge - Technical issues | Technical issues not captured in other codes | 1 mentioned Privacy firewalls | -        one of the thorns in the side, though, is for people at work in finance, or some of those big sectors that have all their privacy firewalls batted down. So, telehealth won't work so but that's you know a minority compared to the bigger picture" - Gc1 |
| TH Challenge - Technical issues - platform specific | Platform specific (Pexip) technical issues | 5 GCs referred to platform specific concerns, 3 voiced the same concern that the pexip platform because it wasn’t a familiar/well known to patients at GCs system, it is prone to more tec issues – getting used to. |  |
| TH Challenge - Technical issues - Wasting time | The loss of time to fix technical issues causes GCs frustration | 5 reported waste of time for dealing with technical issues, adds stress and frustration | " Telehealth it was not just the forgetting but it might work on our end or it might have worked on their end. So you know, start working then it stops or something like that. So it was the both the difficulty with connecting, but also then the frustration and the time it took. And then that might have compromised on the time you have left for the rest of that appointment." - GC 3 |
| TH Benefits - increased rapport | Benefit of TH is increased rapport and increased engagement | 3 GCs commented on increased patient engagement, 2 GCs specifically mentioned that patients were able to feel comfortable in their home and have support persons that would otherwise not be able to be there in-person. | “I think the patients feel more comfortable for those recovering from surgery. You know they don't have to feel worn out having to come to the hospital. They can be comfortable in their lounge or in their bed if they need you. You know, I've seen cups of coffee come in and things like that, so there's a lot more relaxed sort of sense, and I think that gives us now patient experience plus also a more engaged patient” - GC 1 |
| TH Challenge - lack of engagement | Challenge of TH is a lack of engagement | 2 GCs, 1 said more so that recently trend of people forgetting telehealth, 1 found th less engaging | "it is frustrating for me as a clinician to have that unconditional positive regard for a patient, when I feel that they're not even engaged with me or what I'm trying to do for them. So it does, it really challenges me as a genetic counselor to to kind of, you know, be everything that I need to be for that person, when I feel that they're not giving me that same respect back. But that as I said, is is just this, this really small number of people where they just don't seem as engaged or just don't take don't seem to take the telehealth appointment as seriously, as a more formal in-person appointment." - GC 5 |
| TH Challenge - less rapport | Challenge of TH is struggled to build rapport leading to less engagement | 3 GCs made comment on less rapport building in context of usual (i.e. face to face) | "I think there's a there's less chit chat, less umm so I think they often are quicker consults, I think. Yeah, I guess it's harder to build with rapport with them.” – GC 4 |
| TH Challenge - Privacy | Privacy cited as issue that is seen as a challenge when using Th | 4 Gcs commented on privacy, 1 about who is present/influencing responses or just seeing into their space and vica versa | "I worry about, uh, well, sometimes I do have that slight worry that if there is someone else outside of the room that I can't see. I'm wondering what you know influence they have over what's being discussed from the patients point of view." - GC6 |
| TH - GC Suggested Improvements | GCs suggested how TH can be improved to improve usability | One wanted to include function that would allow forms such as consent to be signed over telehealth. 2 GCs thought a more detailed trouble shooting guide for GCs and patients would improve the service as well as a new email link the day before or day of appointment to reduce the likeliness of links getting lost in emails. There were 3 suggestions that were platform specific. |  |
| Phone - challenges | When GCs explicitly associated challenges to phone consults | 1 GC explicitly stated a few phone challenges; lack of visual component, lack of engagement and not being able to connect leading to ‘phone tag’ | *“Phone’s good, but I don't get that sense necessarily that you can always read the non-seen cues, the body language and facial expressions and you know the I guess the reactions to things - you don't get that, and you can’t always tell when you're listening on a phone, what those are.” - GC 1 “in my general experiences, they completely don't value that that's a legitimate appointment with the clinician. I think they feel like it's just, you know, a nice service that we provide after the fact or sort of thing. But I don't think a lot of patients that are booked for phone appointments actually value what it is.” - GC 1* |
