# Supplementary material for: Cancer genetic counseling via telegenetics and telephone: A qualitative study exploring the experience of patients and genetic counselors in an Australian cancer genetics context
Source: J Genet Couns. 2024 Oct 6;34(2):e1982. doi: 10.1002/jgc4.1982 (PMC11953582; doi:10.1002/jgc4.1982)
Supplement: Supplementary file 2 — Appendix S2 [file JGC4-34-0-s002.docx]

| Code | Description | Summary | Quotes |
| --- | --- | --- | --- |
| Choice between modes of delivery | Patients asked out of Telehealth (TH), Telephone (ph) and in-person (F2F) what would they choose in future for GC. | The breakdown: 60% preferred TH (however 3 did outline when it wouldn’t be their choice i.e., if it was more emotional loaded or needed a physical exam), 10% Telephone (ph), 10% in-person (ph) or telehealth. 10% ambivalent. (ph) | “I prefer if I feel like some sort of up issue with my body or some pain, I would prefer to go to the doctor, to complete the examining but if they just the consult, like consult/consultation or asking a question then yeah, I would prefer the telehealth.” - P072.1 (for TH preference)  “In this context, I would choose a phone appointment. I don't, yeah, I see what the video would have added in this context” – P088.  (For phone preference) |
| In-person challenge – Travel/Time commitment | Not directly asked about the challenges of in-person but did ask about stress levels across all 3 models, patients associated travel and time stress with F2F. | 4 patients all reported the stress and commitment associated with in-person consults because of travel commitment and taking day off work. | “I think it would have been more stressful and not necessarily from an interpersonal perspective, but from a have to take the day off, drive to [hospital], have to find parking, have to find the actual office where I'm going to and all of those would have been way more stressful than switching on a computer and trying to get my head around Pexip.” P079 |
| TH Benefit – Visual element | When asked about benefit of TH, they explicitly described how being able to see GC added value. | **5** participants explicitly stated being about to ‘put a face to name’ added a positive value to clinic. | “I feel like I'm at home I can share more. I don't know, but I feel comfortable that I can and I trust the person, like I see the person, I can share more. But in any other way I may just share some general things instead of going into the details.” P072.1 |
| TH Benefit –Saving time/Flexibility | Benefit of TH is not travelling in-person allows for less time to be committed to GC, allowing flexibility | 3 patients directly noted benefit of TH for them was saving time. | Just didn't have to take any time off work like it was, just easy for me to just jump on middle of like my workday and just get it done that way rather than having to get out, get to the car, go and see someone I guess for a rain perspective as well.  P089 |
| TH Benefit –Reduced travel | Benefit of TH is not travelling in-person | 7 patients directly noted TH Benefit was lack of travel. | P078 – more personal and without travel “I would say when it comes to like genetic stuff like this, it's gonna be the best of the three. Yeah, I like it's more personal than a phone call and it's easier than having to travel to the hospital.”  is that quite a significant amount of time out your day or is that quite easy for you just personally? [00:10:06] It tends to be yeah, fair bit of time out of my day. Nowhere is ever easy to get to in Sydney.” P077  P086 – “I think it might be more stressful because of the time factor and the travel. Which is only small but in Sydney, you know, finding a park and all that can be a nightmare. For me it was easier to do it on telehealth.” |
| Th Benefit - Other | Other benefits of TH not falling under the other codes | TH is just as easy and same stress as phone.  Ability to have a support person call in.  Increased engagement on video  Flexibility | P073 – more engagement than phone “I think I pay attention more if I’m on video”.  P079 - “I think I would have; I would have liked to have a support person with me. My husband maybe, and I think telehealth makes that possible, whereas it's completely impossible at the moment for face-to-face appointments.”  P079 - “flexibility. That's what's the most important thing. That you can do it wherever you want to do that, with whomever you want to do, the present like who's there and from time management perspective it's so much better. Because especially if you work Monday to Friday, for a full-time person. |
| TH Challenge – Privacy | When asked if they were worried about anything with TH, some participants cited privacy and security concern | 3 Participants mentioned privacy as a worry but not a worry that was present during the clinic, more so when they think about TH, they are aware of privacy issues. | P077 – I mean, I guess I'm aware that there's potentially privacy things, but it's not, I don't know, it doesn't. [00:11:38] Yeah, it's not really reality that you worry about [00:11:40] Not as significant worry for me. |
| TH Challenge – Technical challenges | TH challenges that include any technical issues from apps to glitches | 3 Participants mentioned technical barriers as challenges e.g., internet issues or app download – one of them reported audio issues in their TH made the consult happen over the phone – but was fine with the switch | what do you think might be the difficulties of using telehealth for you? [00:05:42] I guess you just have to make sure, it's the same, you just have to make sure you have the Internet connection.  P073 |
| TH Challenge – Other | Reponses to difficulties of using telehealth that do not fall under the other codes | Answers (4 responses) range from lack of human connection to lack of physical connection. | if it was more of like a physical medical appointment. It wouldn't apply and it would probably be a waste of time to have a telehealth first and then have to go in. If at, and if at the end of this appointment I have to go in, I prefer to just go in in the first place.  P078 |
| Phone benefit – saving time flexibility | When about benefits of phone consults, perceived time saver, reduction of travel time and tec set up | 1 mentioned benefit of phone was more efficient | **“So, I think that’s probably a big benefit for you guys, but also from us just to have that convenience of being able to jump on. You know, in the middle of our day, and not have to schedule the whole day around that one specific appointment is like, yeah, huge benefit.”**  **P089** |
| Phone benefit – other | Reponses to benefits of using phone that do not fall under the other codes | 3 reported.  1 was no glitches.  1 easier than TH  1 decrease in stress and ability to move around | P079 – technical a lot easier but lack of seeing “I think, I think technically it would, it's a lot easier to use the phone. Cause everyone can use the phone”.  P088 - the fact that it was a phone appointment like made things better because I don't have to stress about getting to the hospital on time. Yeah, or anything like that. I would have obviously taken the video call if that's what was given, but I do prefer the phone because I can put my earphones in and I can move around if I want to, it's just a bit easier. |
| Phone challenge – not seeing face | When about difficulties of phone consults, not having visual factor | 1 reported | P079 - “I think, I think technically it would, it's a lot easier to use the phone. Cause everyone can use the phone, but I think from a quality of appointment perspective, I think seeing someone, especially when you talk about family trees and stuff. I think that's a lot easier. And I also think that body language is really important in sensitive conversations, and I think you've missed that completely on the phone.” |
| Experience | Their overall genetic counselling experience whether phone to TH | All reported good experience. I reported a 9/10 experience.  Only 1 reported apprehension – a phone stream participant felt apprehension about having appointment over the phone.  All reported GC was easy to talk to. | P079 –TH - I thought it was great. I think, I think an appointment like that way you don't actually need to be hands on at all and where it's just the chat is perfect for telehealth, I think.  “So, before I had the appointment I would have said, I definitely prefer a videocall, but the phone call was fine.” - P077  P088 – “It was super easy; the counsellor was really friendly and informative, and she explained everything really deeply.” |
| Confidence with IT | Asked to describe their confidence with IT | All reported above average. The lowest descriptor being 6/10 and the highest descriptor being very high confidence |  |
| Past experience with TH | Answers to have they used telehealth and then if they have used videoconferencing technologies. | 3 have had telehealth experience before. All have had experience with videoconferencing, for work and social contexts. |  |
| Organising appointment | Asked if they had to organise anything for appointment | All reported they did not have to organise anything, 4 mentioned they were able to do on break at work. | “It didn't take any, you know, time out of your day. Uh no, you know, because I have one hour break during the day. So, I guess we use that one.” P072.1 |
| Other – Suggested improvement | Asked for any suggested improvement across their delivery model. | 1 participant had suggestion to improve TH experience – to send link day or morning before appointment to avoid digging through emails for link. |  |
| Support – IT | When asked what support they would want from service to use TH | Majority could not think of anything, 3 phone stream participants mention they would want IT support, 1 of them said not specifically for them but as standard service and 2 said platform specific instructions would be what they expect. | “I suppose just kind of instructions on which platform to use and then just basic instructions on how to connect if there are any special instructions. Otherwise, it goes fairly standard than just which platform to use.” |
| COVID | When participant would mention COVID-19 pandemic | 8 participants mentioned COVID – mainly in relation to their experience with videoconferencing technologies in the context of work | “No, probably started with COVID. Everything used to be face to face, but yeah, it's just a normal yeah” P089.  I'm not sure if it's because I'm used to it because of these two years of the COVID, but yeah, I can just talk about like now, right now, I prefer video call then it calling just before a phone.  P072.1 |
