# Supplementary figures and images for: Cancer genetic counseling via telegenetics and telephone: A qualitative study exploring the experience of patients and genetic counselors in an Australian cancer genetics context
Source: J Genet Couns. 2024 Oct 6;34(2):e1982. doi: 10.1002/jgc4.1982 (PMC11953582; doi:10.1002/jgc4.1982)

**Supporting information**

Interview Guide


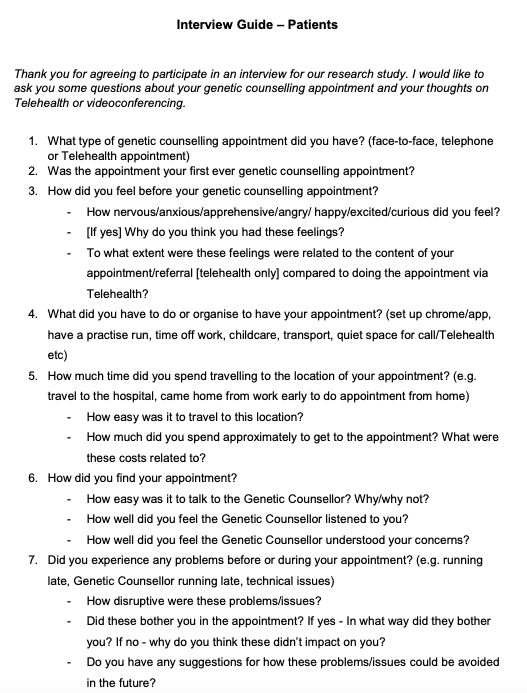


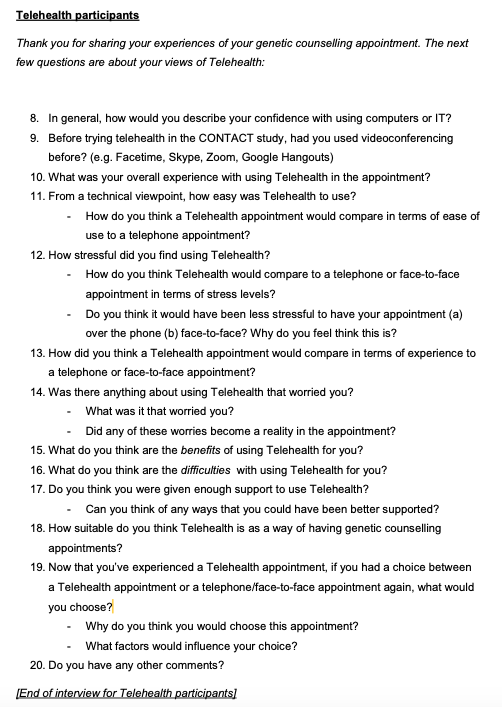


**
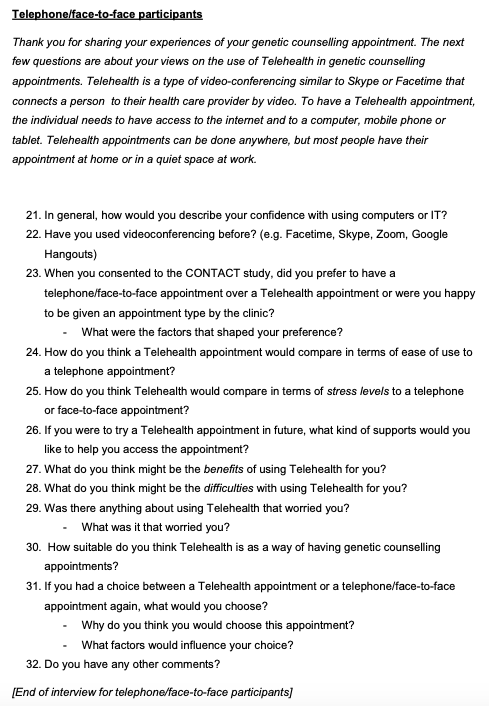
**


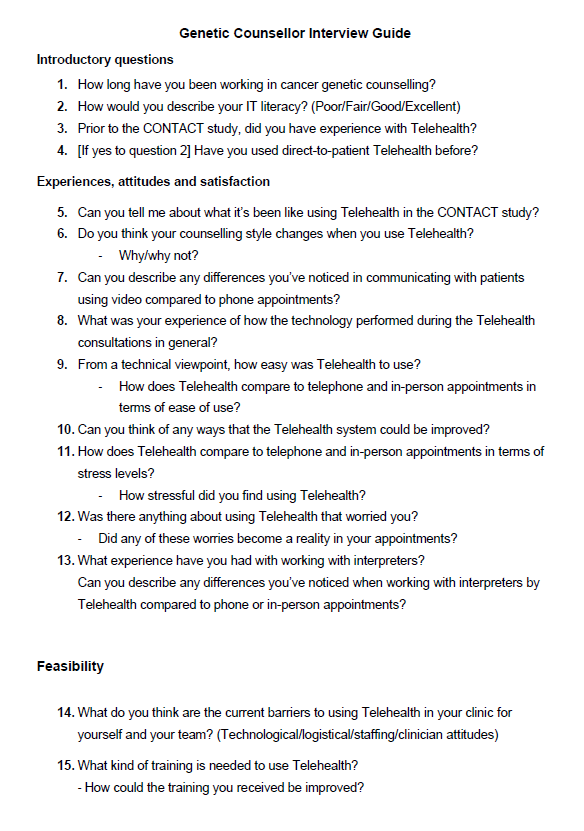


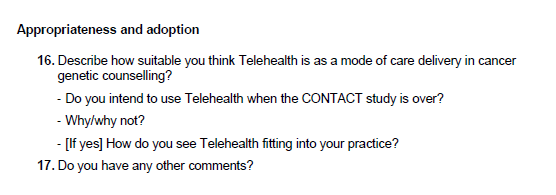

Supplement: Supplementary file 3 — Appendix S3 [file JGC4-34-0-s003.docx]
